# Frequency of Pathogenic Paediatric Bacterial Meningitis in Mozambique: The Critical Role of Multiplex Real-Time Polymerase Chain Reaction to Estimate the Burden of Disease

**DOI:** 10.1371/journal.pone.0138249

**Published:** 2015-09-22

**Authors:** Aquino Albino Nhantumbo, Vlademir Vicente Cantarelli, Juliana Caireão, Alcides Moniz Munguambe, Charlotte Elizabeth Comé, Gabriela do Carmo Pinto, Tomás Francisco Zimba, Inácio Mandomando, Cynthia Baltazar Semá, Cícero Dias, Milton Ozório Moraes, Eduardo Samo Gudo

**Affiliations:** 1 Laboratório Nacional de Referência de Microbiologia, Instituto Nacional de Saúde, Ministério da Saúde, Maputo, Mozambique; 2 Universidade Feevale, Rio Sul, Brazil; 3 Universidade Federal de Ciências de Saúde de Porto Algre (UFCSPA), Porto Alegre, Brazil; 4 Laboratório de Isolamento Viral, Instituto Nacional de Saúde, Ministério da Saúde, Maputo, Mozambique; 5 Departamento de Medicina at the Hospital Central de Maputo, Ministério da Saúde, Maputo, Mozambique; 6 Centro de Investigação em Saúde da Manhiça, Ministério de Saúde, Maputo, Mozambique; 7 Fundação Oswaldo Cruz, Rio de Janeiro, Brazil; 8 Instituto Nacional de Saúde, Ministério da Saúde, Maputo, Mozambique; London School of Hygiene and Tropical Medicine, UNITED KINGDOM

## Abstract

**Background:**

In Sub-Saharan Africa, including Mozambique, acute bacterial meningitis (ABM) represents a main cause of childhood mortality. The burden of ABM is seriously underestimated because of the poor performance of culture sampling, the primary method of ABM surveillance in the region. Low quality cerebrospinal fluid (CSF) samples and frequent consumption of antibiotics prior to sample collection lead to a high rate of false-negative results. To our knowledge, this study is the first to determine the frequency of ABM in Mozambique using real-time polymerase chain reaction (qPCR) and to compare results to those of culture sampling.

**Method:**

Between March 2013 and March 2014, CSF samples were collected at 3 regional hospitals from patients under 5 years of age, who met World Health Organization case definition criteria for ABM. Macroscopic examination, cytochemical study, culture, and qPCR were performed on all samples.

**Results:**

A total of 369 CSF samples were collected from children clinically suspected of ABM. qPCR showed a significantly higher detection rate of ABM-causing pathogens when compared to culture (52.3% [193/369] *versus* 7.3% [27/369], *p* = 0.000). The frequency of *Streptococcus pneumoniae*, *Haemophilus influenzae*, group B Streptococci, and *Neisseria meningitidis* were 32.8% (121⁄369), 12.2%, (45⁄369), 3.0% (16⁄369) and 4.3% (11⁄369), respectively, significantly higher compared to that obtained on culture (*p* < 0.001 for each).

**Conclusion:**

Our findings demonstrate that culture is less effective for the diagnosis of ABM than qPCR. The common use of culture rather than qPCR to identify ABM results in serious underestimation of the burden of the disease, and our findings strongly suggest that qPCR should be incorporated into surveillance activities for ABM. In addition, our data showed that *S*. *pneumoniae* represents the most common cause of ABM in children under 5 years of age.

## Introduction

Acute bacterial meningitis (ABM) is the most severe and potentially fatal type of meningitis. Despite the introduction of a vaccine for *Haemophilus influenzae type b* (*Hib*), ABM still represents a significant cause of childhood morbidity and mortality in Sub-Saharan African [1,2]. In these settings, the patient fatality rate is high, reaching up to 31.3% in children under 5 years old [3]. Early diagnosis and rapid intervention, including antibiotic therapy, are critical to improve patient outcomes [4,5].

In Sub-Saharan Africa and other regions, ABM is mainly caused by a triad of species: *S*. *pneumoniae*, *H*. *influenzae*, and *N*. *meningitidis*, [1,6,7]. In Mozambique, information regarding burden of childhood bacterial meningitis is scarce. Only 2 studies have been performed in southern Mozambique [6,7]. However, Mozambique is a large country and because of the spatial, environmental, cultural, demographical and socio-economical differences between regions, the results of these studies are difficult to generalise to the entire paediatric population in Mozambique since they were conducted in southern Mozambique and no data is available for other regions of the country, In addition, both studies were performed before 2009, more than 6 years prior to our study, and don’t reflect the recent impact of *Hib* in the epidemiology of ABM. Understanding the epidemiology of ABM in other regions of the country and the recent impact of *Hib* vaccinations is critical for public health interventions.

The emergence of antimicrobial resistance, especially multiple drug resistance, has added another layer of complexity to this problem and little information is available in Mozambique. The little available data on antimicrobial resistance were obtained from studies conducted in the southern Mozambique [2,6].

Microbiological culture of cerebrospinal fluid (CSF) is considered the gold standard for the diagnosis of ABM and it is time-consuming. In addition, the sensitivity of this method is seriously hampered by low quality CSF samples and use of antibiotics prior to the lumber puncture, both of which are common in resource-limited settings [8], such as Mozambique [6]. In fact, several studies conducted in Sub-Saharan Africa demonstrated that the use of culture resulted in under-diagnosis and underestimation of the burden of bacteria involved in the aetiology of ABM [9,10,11].

Molecular testing based on real-time polymerase chain reaction (qPCR) has been strongly proposed as an alternative diagnostic tool to overcome and address the low sensitivity and other methodological disadvantages of microbiological culture in resource-limited settings [10,12–14]. Previous reports demonstrated that molecular testing significantly increased the detection rate of bacteria involved in the aetiology of ABM [9–15].

Early and effective laboratory confirmation of the aetiology of ABM is critical not only for patient care but also for monitoring of the impact of routine meningitis vaccination [16–18]. In Mozambique, *Hib* conjugate vaccine and pneumococcal conjugate vaccine (PCV-10) were introduced in 2009 and 2013, respectively, and implementation of laboratory-based surveillance to monitor the impact of these vaccines is critical. In Mozambique, surveillance for ABM is weak, traditionally concentrated in the southern region, and mainly based on culture sampling. Recently, the National Microbiology Reference Laboratory expanded ABM surveillance to other two regions (central and northern) and also implemented qPCR.

This study was conducted to determine and compare the frequency of pathogens involved in the aetiology of ABM using microbiological culture, multiplex qPCR (M-qPCR), and the corresponding antibiotic susceptibility test in 3 regions in Mozambique.

## Materials and Methods

### Study sites

This study was conducted in 3 quaternary hospitals in Mozambique, namely Hospital Central de Maputo (HCM), Hospital Central da Beira (HCB), and Hospital Central de Nampula (HCN), situated in the southern, central, and northern regions of the country, respectively. The HCN is located in Nampula province. Nampula province has 23 districts and a total of 3,985,613 inhabitants [19]. The pediatric guard at this hospital has 184 beds. The HCB is located in Sofala province which has 13 districts, and a total of 1,642,920 inhabitants [19]. The pediatric guard at this hospital has 150 beds. The HCM is located in Maputo city, which has 8 districts, and a total of population of 1,766,823 inhabitants [19]. This hospital has 322 beds in the pediatric guard. All of them are reference hospitals for their region and offer several specialised services for all age groups.

Mozambique has a subtropical climate with 2 distinct seasons: the rainy season from November and April, and the dry season from May to October.

### Ethics statement

The study was approved by the Mozambican National Bioethics Committee (Ref #: IRB00002657). Verbal consent to participate was obtained from the legal representative of each child, as this study was conducted as part of the routine sentinel surveillance for acute bacterial meningitis being implemented by the Ministry of Health in Mozambique. In this surveillance, only verbal consent is routinely requested and approved by National Bioethics Committee. Each participant who provided verbal consent was recorded in a logbook at the health facility.

### Study design and case definition

Between March 2013 and March 2014, a cross-sectional study was implemented as part of the recently expanded sentinel surveillance for paediatric ABM in Mozambique. Children admitted at each of these sentinel sites who met World Health Organization (WHO) case definition for ABM were consecutively enrolled. As per WHO, suspected case of ABM was defined as a child aged < 5 years with sudden onset of fever (>38.5°C rectal or 38.0°C axillary) and 1 of the following signs: neck stiffness or flaccid neck, bulging fontanel, convulsion, irritability, or drowsiness. Request of verbal consent to participate in this study, clinical examination, lumbar puncture and the fill of the ABM case investigation form were performed by the paediatrician attending the admitted child.

### Sample collection and investigation form

CSF samples were aseptically obtained from each participant through lumber puncture (LP). Up to 1 mL of CSF was collected into each of 2 3.0 ml sterile tubes. Samples were immediately sent to the local laboratory for microbiological analysis including cytochemical study. CSF was considered purulent when at least 1 of the following criteria were met: i) turbid/cloudy appearance, as assessed by the physician; ii) leukocyte count ≥100 mm^3^; or iii) leukocyte count between 10–100 mm^3^ and either a glucose level <40 mg/dL or presence of protein as determined using a semi-quantitative method (Pandy).

On admission, clinical and demographic data, vaccination, and therapeutic history was collected by a trained medical officer using a standard case investigation form.

### Laboratory methods

#### Laboratory at the sentinel site

At the local laboratory, the CSF samples collected into the first tube was used to measure glucose, Gram staining, and bacterial culture. The CSF collected into the second tube was used to perform cell count and protein measurement (with use of Pandy, a semi-quantitative method). All negative and positive CSF samples, as well as all isolates, were sent to the National Reference Microbiology Laboratory (NRML) at National Institute of Health (NIH) for bacteriological confirmation, assessment of antibiotic susceptibility profile by characterisation of Minimal Inhibitory Concentration (MIC), and qPCR.

#### National Reference Laboratory

At the NRML, isolates were recovered by plating onto 5% sheep blood and chocolate agar plates (MAST, Merseyside, UK). Twenty-four hours after incubation at 35° C ±2°C with 5% of CO_2_, bacterial isolates were identified by colony morphologic analysis and growth requirement. Pneumococci were identified based on morphological features in Gram stain, optochin susceptibility test (OXOID–DD1 OPTOCHIN, Basingstoke, England), and bile solubility (BD–BBL Desoxycholate Reagent Droppers, Becton Dickinson and Company, USA) [20]. *Neisseria meningitides* species were identified by colony morphology, Gram stain, oxidase test and carbohydrate utilization (glucose, maltose, lactose and sucrose) using cysteine trypticase agar (CTA) method while *Haemophilus influenzae* identification was based on X and V growth factors. *H*. *influenzae* was grown around the paper disk containing both haemin (factor X) and NAD (factor V). *Streptococcus agalactiae* was identified by the morphology of the colony, Gram stain and CAMP test. After identification, isolates were subsequently stored in 20% skim milk at -80°C for further investigation.

### Antimicrobial susceptibility test

An antibiotic susceptibility test of each isolate was performed using the disk diffusion method and results were interpreted according to M100—S22 document of the Clinical and Laboratory Standards Institute Performance Standards for Antimicrobial Susceptibility Testing [21]. For pneumococci, penicillin susceptibility was first screened using oxacillin disk diffusion. For all isolates resistant to oxacillin (halo diameter <19mm), MIC to penicillin was determined by E-test^®^ (Biomerieux, SA). All isolates were also tested for erythromycin, vancomycin, trimethoprim-sulfamethoxazole, tetracycline, and ceftriaxone. We also determined antibiotic susceptibility for levofloxacin and chloramphenicol. For *H*. *influenzae* and meningococci, susceptibilities for chloramphenicol (30 μg), ceftriaxone (30 μg), and ampicillin (10 μg) were determined.

#### DNA extraction

Two hundred microliters of CSF samples were used for DNA extraction. DNA was extracted using either Biopour mini kit (BIOPUR, Biometrix diagnostic, Brazil), or High pure PCR template kit (Roche Diagnostics Corporation, US) according to the manufacturer’s instructions. Total DNA was eluted in 200 μl of appropriated elution buffer and stored at −20°C until use.

### M-qPCR

M-qPCR was performed using a set of specific primers for simultaneous detection of the following target genes: capsular transport for *Neisseria meningitidis*, pneumolysin (*ply*) for *Streptococcus pneumoniae*, *bex A* for *Haemophilus influenzae* and *cfb* gene for *Streptococcus agalactiae* (group B streptococci), as described by Corless *et al*., 2001 [14]; Ke *et al*., 2000 [22] and El Aila *et al*., 2011 [23].

This protocol was based on SYBER detection system (Platinum SYBR Green qPCR Supermix-UDG, Invitrogen, California, UK) using the LightCycler 2.0 instrument (Roche Diagnostic GmbH, Mannheim, Germany). Primers were used at 10 pM each. The mix included 10 μl of Platinum SYBR Green qPCR Supermix-UDG, 1 μl of each primer (*S*. *pneumoniae*, *H*. *influenzae*, *N*. *meningitidis* and *S*.*agalactiae*), 3 μl of sterile PCR grade water and 2μl of template DNA were added in a final volume of 20 μl. Negative controls consisting of PCR grade water instead of the target DNA (2 μl per reaction) were used in each assay as well as positive controls of standard strains used for species identification such as *S*. *pneumoniae* ATCC 49619, *H*. *influenzae* ATCC 49247, *N*. *meningitidis* ATCC 13077 and *S*. *agalactiae* ATCC 27591. Samples were amplified as follows: an initial denaturation step at 95°C for 5 min, 2 minutes at 50°C for glycosylase reaction followed by 38 cycles at 96°C for 10 seconds (denaturation), 50°C for 10 seconds (annealing) and 72°C for 8 seconds (elongation). After amplification, melting curve analyses was performed to differentiate the four bacterial pathogens. Melting temperature for *S*. *agalactiae*, *S*. *pneumoniae*, *N*. *meningitidis*, and *H*. *influenzae* were 79.09°C, 79.75°C, 82.93°C, and 84.16°C, respectively.

### Statistical analysis

Data were entered into a database developed using Epi Info version 3.5.4 (IBM, US), and analysed using SPSS statistical software version 20 (CDC, US). Categorical variables were reported as proportion and were compared using Pearson Chi squared test.

Logistic regression model was used to determine the variables associated with ABM. Variables were included in the initial model if the *p*-value in the bivariate analysis was less than <0.25. Backward stepwise logistic regression model was used to select variables for the final model. Variables with the *p*-value less than 0.05 on multivariate logistic regression analysis were considered to have a statistically significant association with bacterial meningitis infection. Odds ratios (OR) and 95% confidence interval [95% CI] were computed.

## Result

### Demographic characteristics

**Fig 1:**
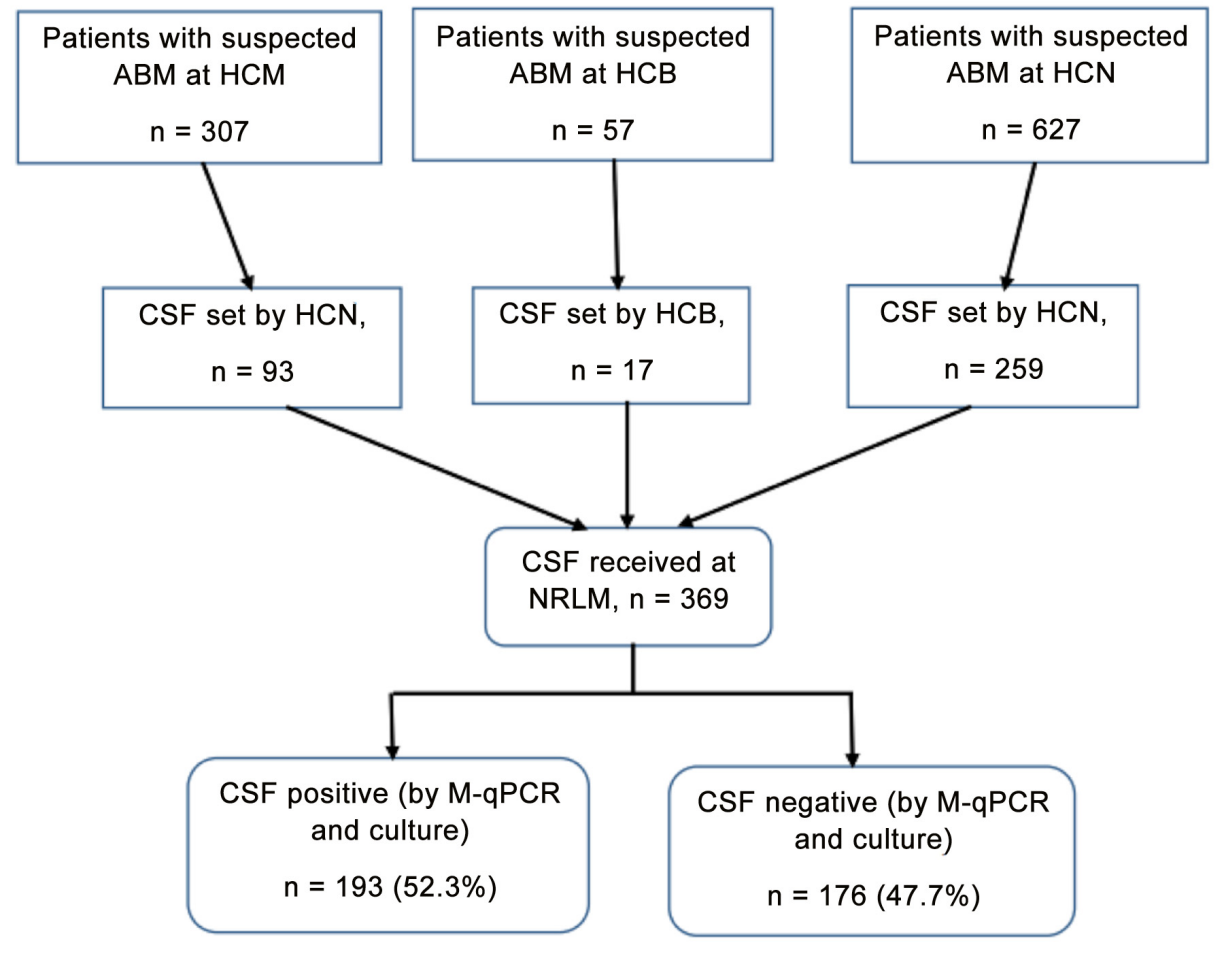
Numbers of patients enrolled and tested. The flow chart show the number of children under 5 years old who were admitted at each hospital during the study period. ABM: acute bacterial meningitis; CSF: Cerebrospinal fluid; HCB: Hospital Central da Beira; HCM: Hospital Central de Maputo; HCN: Hospital Central de Nampula; M-qPCR: multiplex real-time polymerase chain reaction; NMRL: National Reference Microbiology Laboratory

CSF samples from a total of 369 children under 5 years of age, who were clinically suspected of ABM were received and tested at the NMRL. Of the 369 children, 193 (52.3%) were male. The median age was 9 months (IQR: 0–59 months). Of the 369 samples, 93 (25.2%) were from HCM, 17 (4.6%) from HCB and 259 (70.2%) from HCN (Fig 1).

### Sybr Green-based qPCR increased detection rate of bacterial meningitis

Table 1 compares the performance of microbiological culture against qPCR for the detection of ABM in CSF samples. Bacterial pathogens causing ABM were detected by M-qPCR in 52.3% (193/369) of CSF samples, while only 7.3% (27/369) of CSF samples were culture-positive. This represents a seven-fold increase in the detection rate of ABM.

**Table 1:** Performance comparison of culture against multiplex real-time polymerase chain reaction (M-qPCR).

Test performed	RT-PCR+	RT-PCR-	Total
**Culture +**	27	0	27
**Culture -**	166	176	342
**Total**	193	176	369

In the present study, microbiological culture was occasionally performed immediately, but was instead usually delayed for up to 2 hours, which potentially decreased the sensitivity of culture. Moreover, the consumption of antibiotic prior to lumbar puncture may have decreased the sensitivity of culture. For this reason, culture was not considered the gold standard method in this study. Results of culture were compared to M-qPCR. Using qPCR as the gold standard, the sensitivity of culture for the detection of ABM was very low (14%). The specificity of culture was high (100%, data not shown), but qPCR was able to identify several cases that had not been detected by culture (see Table 1). Positive and negative predictive values of culture were 100% and 47.7% respectively. The agreement between the two tests was weak (*kappa* = 0.134) and was statistically significant (*p* = 0.000) using *McNemar* test.

Results of culture obtained at the sentinel were compared to those obtained at the NMRL and we found that the agreement was 100% for each pathogen (*p* = 0.998) (see Table 2).

**Table 2:** Comparison of the results of culture between local laboratory and NRML.

	Bacteria causing ABM				
Local	*S*. *pneumoniae* (n = 17)	*H*. *influenzae* (n = 5)	*N*. *meningitidis* (n = 3)	*S*. *agalactiae* (n = 2)	*p value*
**NRLM**	17(100%)	5(100%)	3 (100%)	2 (100%)	0.998
**Local Site**	17 (100%)	5 (100%)	3 (100%)	2 (100%)	

*Streptococcus pneumoniae* was the most commonly detected bacteria (4.5%, 17/369) when using microbiological culture, followed by *H*. *influenzae* (1.4%, 5/369), *N*. *meningitidis* (0.8%, 3/369), and group B streptococci (0.5%, 2/369).

**Fig 2:**
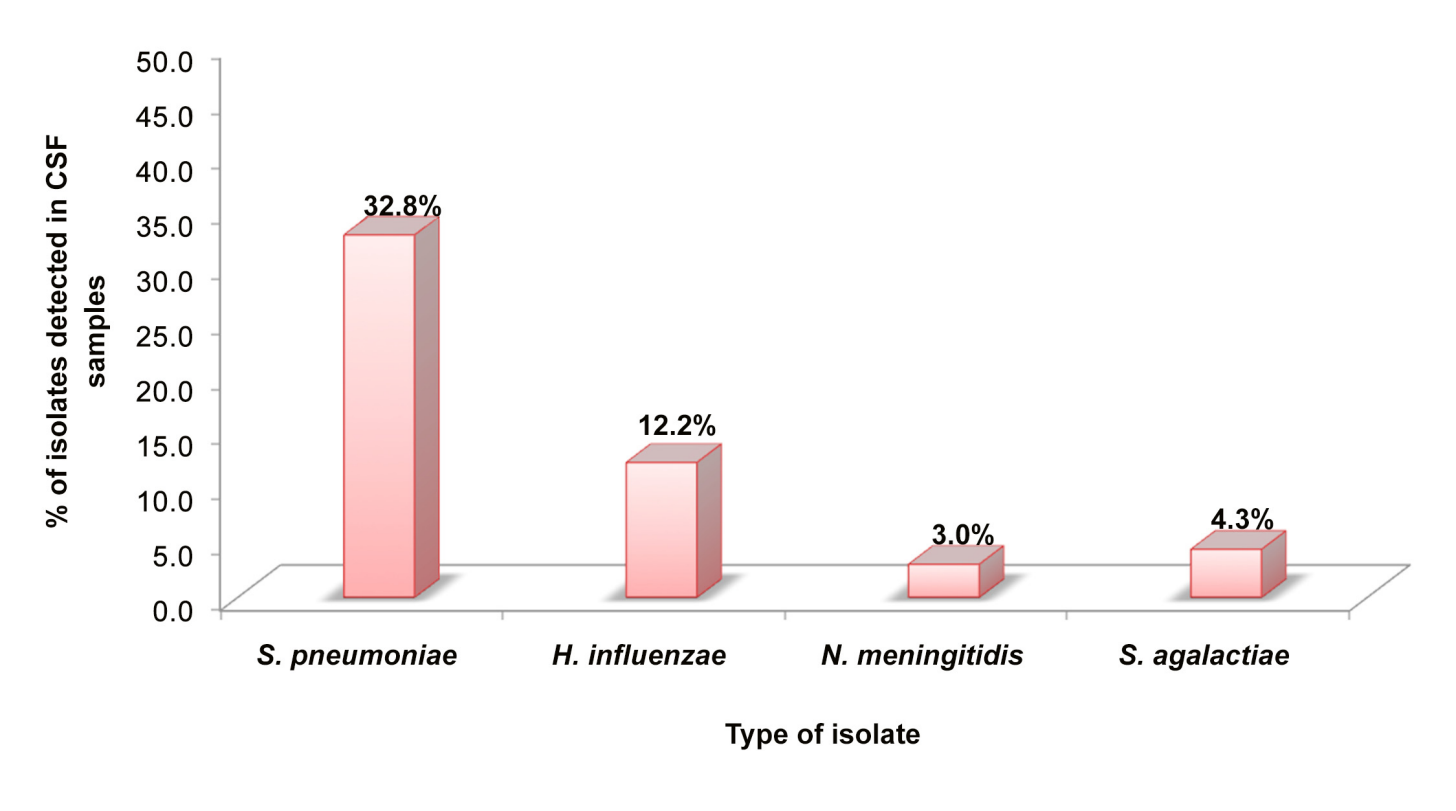
Proportion of pathogens causing acute bacterial meningitis cases in Mozambique in the period between March 2013 and March 2014. This figure show the proportion of pathogens causing ABM in Mozambique in the period between March 2013 and March 2014 using multiplex real-time polymerase chain reaction (M-qPCR). ABM: Acute Bacterial Meningitis.

Similarly, in the M-qPCR, *S*. *pneumoniae* was also the most commonly detected bacteria in the CSF samples (32.8%, 121/369), followed by *H*. *influenzae* (12.2%, 45/369). Therefore, using this method, the least frequent cause of ABM was determined to be *N*. *meningitidis*, which was identified in 3.0% (11/369) of samples, following group B streptococci, which was identified in 4.3% (16/369) of samples (Fig 2).

Table 3 demonstrates that the main cause of ABM was distinct in different age groups. While in children aged less than 1 month old, group B streptococcus was the most common pathogen causing bacterial meningitis (90.9%; 10/11), in children between 1 month and 5 years old, *S*. *pneumoniae* was the most common *(*66.5%, 121/182), followed by *H*. *influenzae* (24.2%, 44/182), and *N*. *meningitidis* (6.0%, 11/182) (see Table 3).

**Table 3:** Age stratification of acute bacterial meningitis (ABM) patients based on multiplex real-time polymerase chain reaction (M-qPCR).

		Bacteria causing ABM			
		*S*. *pneumoniae*	*H*. *influenzae*	*N*. *meningitidis*	*S*. *agalactiae*
Age group (month)	No. of confirmed ABM	n (%)	n (%)	n (%)	n (%)
**<1**	11	0 (0)	1(9.1)	0 (0)	10 (90.9)
**1–11**	124	78 (62.9)	35 (28.2)	6 (4.8)	5 (4.0)
**12–23**	25	19 (76.0)	2 (8.0)	4 (16.0)	0 (0)
**24–59**	33	24 (72.7)	7 (21.2)	1 (3.0)	1 (3.0)
**Total**	193	121 (62.7)	45 (23.3)	11 (5.7)	16 (8.3)

### Geographical and seasonal variability in the frequency of ABM

The number of patients enrolled at the three sites was different and for this reason, absolute numbers of ABM were not compared, instead we compared the percentages (relative frequencies). Our results showed that the relative frequencies of ABM at the three sites were similar (52.9%, 137/259, in HCM in the northern, 50.5%, 47/93 in HCM in the southern, and 52.9%, 9/17 in HCB in central Mozambique), although the relative frequency at HCM was slightly lower.

**Fig 3:**
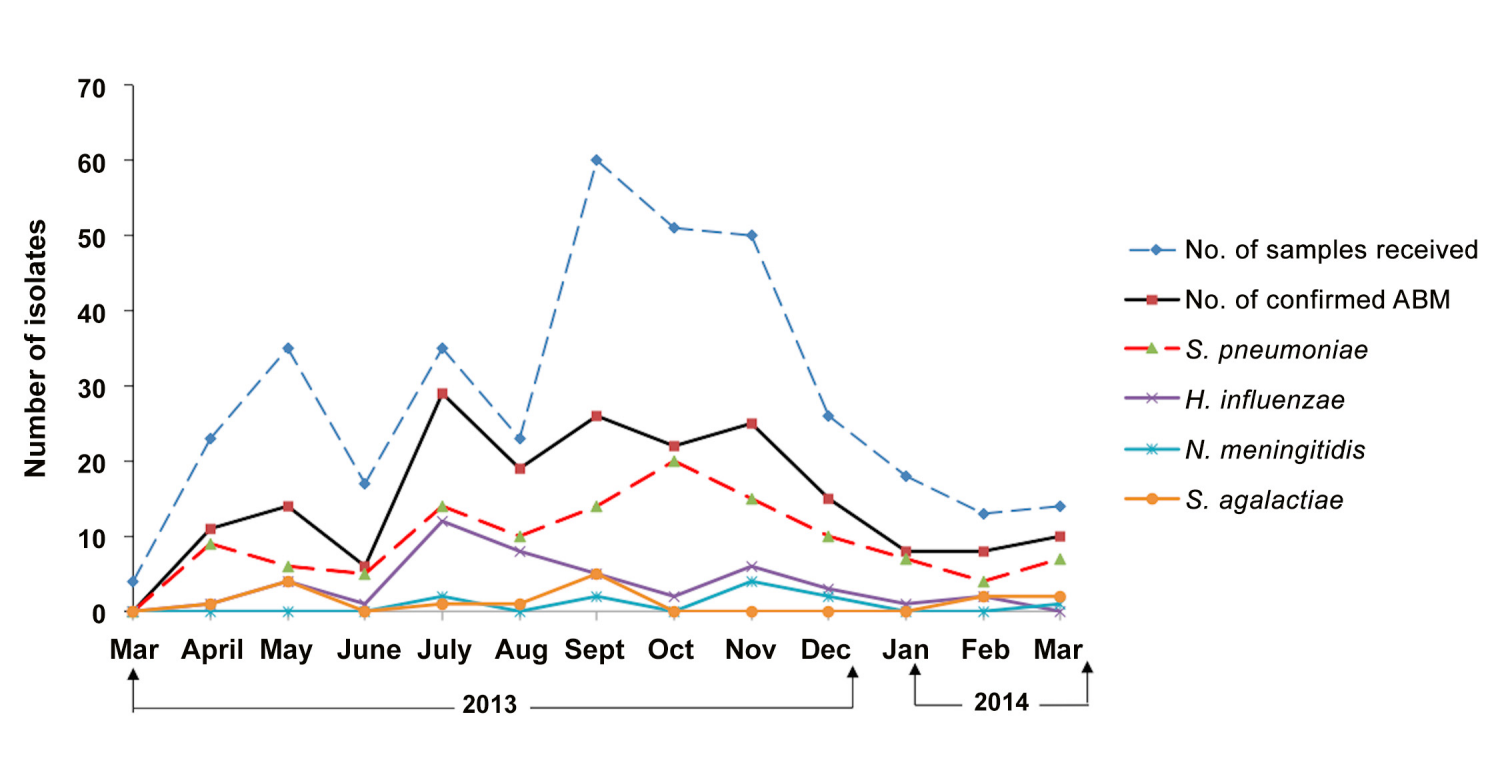
Monthly variation of the relative frequency of pathogens causing ABM. This figure show the monthly variation of relative frequency of pathogens causing ABM and also the variation in the number of CSF samples collected from children <5 years. Frequency of pathogens was determined using multiplex real-time polymerase chain reaction (M-qPCR). ABM: acute bacterial meningitis; CSF: cerebrospinal fluid

Fig 3 shows monthly variation in the recruitment of children fulfilling the definition criteria for ABM in the 3 sentinel sites and demonstrates an increase of suspected and confirmed cases of ABM, mostly in the dry season, peaking in July, September, and November.

### Predicting variables associated with ABM infection

Table 4 represents the analysis of variables that could be used to predict ABM based on M-qPCR results.

**Table 4:** Univariate and multivariate analyses of demographic and laboratory variables associated with acute bacterial meningitis (ABM) infection in children under 5 years of age in Mozambique based on multiplex real-time polymerase chain reaction (M-qPCR). Note: CI: confidence interval; CSF: cerebrospinal fluid; OR: odds ratio

Risk factor	ABM (n = 193)	CSF negative (n = 176)	Unadjusted OR [95% CI]	*p*-value	Adjusted OR [95% CI]	*p*-value
**Age groups, months**						
<1	11 (5.7)	0 (0)	0.0	0.999		
1–11	124 (64.2)	73 (41.5)	3.4 [2.1–5.4]	<0.0001	3.9 [1.7–9.0]	0.002
12–23	25 (13.0)	18 (10.2)	2.7 [1.3–5.5]	0.006	3.3 [0.2–11.9]	0.066
24–59	33 (17.1)	85 (48.3)	1			
**Sex**						
Male	96 (49.70)	97 (55.1)	1			
Female	97 (50.3)	79 (44.3)	1.2 [0.8–1.9]	0.302		
**CSF appearance**						
CSF clear	36 (18.7)	66 (37.5)	1			
CSF turbid	154 (79.8)	102 (58.0)	2.9 [1.8–4.5]	<0.0001	0.8 [0.4–2.0]	0.878
Bloody	1 (0.5)	0 (0)				
Missing cases	1 (1.0)	8 (4.5)				
**Proteins**						
Negative	26 (13.5)	61 (34.7)	1			
Positive	75 (38.9)	27 (15.3)	3.5 [2.1–5.8]	<0.0001	1.9 [2.1–3.5]	0.035
Missing cases	92 (47.7)	88 (50.0)				
**Leukocyte count, cells/mm** ^**3**^						
<10	30 (15.5)	85 (48.3)	1			
10–100	67 (34.7)	33 (18.8)	6.8 [3.0–10.4]	<0.0001	8.7 [3.6–21.1]	0.000
>100	92 (47.7)	17(9.7)	17.6 [3.0–10.4]	<0.0001	26.7 8.9–79.1]	0.000
Missing cases	4 (2.1)	41 (23.3)				
**CSF glucose level, mg/dL**						
>40	2 (1.0)	9 (5.1)	1			
<40	49 (25.4)	4 (2.3)	14.1 [4.9–39.9]	<0.0001	18.6 [3.5–97.7]	0.001
Missing cases	142 (73.6)	163 (92.6)				
**Gram Stain results**						
Positive	137 (71.0)	56 (31.8%)	1.4 [1.28–1.54]	<0.0001	0.0	0.000
Negative	56 (29.0)	120 (68.2%)	1			
**Hospital**						
HCB	9 (4.7)	8 (4.6)	1			
HCM	47 (24.3)	46 (26.1)	1.1 [0.4–3.81]	0.855		
HCN	137 (71.0)	122 (69.3)	1.0 [0.7–1.77]	0.696		

Multivariate logistic regression analysis demonstrated that ABM was statistically significant and associated with age below 12 months, using the age group of 24–59 months as the reference (adjusted OR (aOR) = 3.9; *p* = 0.002), positivity for proteins in the CSF (aOR = 1.9; *p* = 0.03), leukocyte count 10–100 cells/mm^3^ (aOR = 8.7; *p* = 0.000), leukocyte count >100 cells/mm^3^ (aOR = 26.7; *p* = 0.000) and glucose measurement < 40 mg/dL (aOR = 18.6; *p* = 0.001) (see Table 4).

No association was found between ABM and geographical regions, although there was slight trend towards higher frequency of ABM in the northern Mozambique.

### Antibiotic resistance profile

Table 5 summarises the antibiotic susceptibility profile for the 4 bacterial species isolated in this study and demonstrated that all isolates of *S*. *pneumoniae* (n = 17) were susceptible to ceftriaxone and levofloxacin and resistant to trimethoprim/sulfamethoxazole (SXT). Different resistance profile levels were observed for penicillin (88.2%), vancomycin (11.8%), erythromycin (23.5%), tetracycline (64.7%), and chloramphenicol (35.3%).

**Table 5:** Antimicrobial susceptibility profile of acute bacterial meningitis (ABM) isolates from cerebrospinal fluid (CSF). Note: R: Resistant; S: Susceptible; (-): Antimicrobial susceptibility test not performed

	*S*. *pneumoniae* (n = 17)		*H*. *influenzae* (n = 5)		*N*. *meningitidis* (n = 3)		*S*. *agalactiae* (n = 2)	
Antimicrobial agent	R (%)	S (%)	R (%)	S (%)	R (%)	S (%)	R (%)	S (%)
Penicillin G	88.2	11.8	-	-	-	-	-	-
Vancomycin	11.8	88.2	-	-	-	-	100	0
Tetracycline	64.7	35.3	-	-	-	-	-	-
Trimethoprim/Sulfamethoxazole	100	0	-	-	-	-	-	-
Erythromycin	23.5	76.5	-	-	-	-	0	100
Levofloxacin	0	100	-	-	-	-	0	100
Ceftriaxone	0	100	0	100	0	100	0	100
Chloramphenicol	35.3	64.7	20	80	33.3	66.7	-	-
Ampicillin	-	-	0	100	33.3	66.7	100	0
Clindamycin	-	-	-	-	-	-	0	100

All isolates of *H*. *influenzae* (n = 5) were susceptible to ampicillin and ceftriaxone and only one was resistant to chloramphenicol (20%).

Among the meningococci isolates (n = 3), only one was resistant to ampicillin and chloramphenicol (33.3% for each), but all were susceptible to ceftriaxone.

All isolates of group B streptococci (n = 2) were susceptible to erythromycin, levofloxacin, and ceftriaxone, and all were resistant to vancomycin and ampicillin.

## Discussion

This study provides the first ever description of the aetiology of ABM in the 3 geographical regions in Mozambique, namely northern, central, and southern. This represents a significant increase in whole-country representation over previous reports, since previous studies were all conducted in southern Mozambique [2,7]. Although only 1 health facility in each region was selected for our study, they are the reference hospitals for each region. The importance of this study is increased by the fact that for the first time a molecular method based on M-qPCR was used to improve the detection rate on initially cultured CSF samples. Since all samples were paired and tested by culture and M-qPCR, we were able to compare their performance, and results from this study showed a seven-fold increase in the detection rate of ABM when using M-qPCR as compared to culture (52.3% versus 7% respectively). The agreement between culture and qPCR was poor. This is in agreement with previous studies [24,25]. Main reason for these discrepancies, include poor quality of CSF samples and a high rate of consumption of antibiotics prior lumbar punction [8,10,13]. Thus, M-qPCR showed that the burden of paediatric bacterial meningitis in Mozambique is still very high. The frequency is much higher than that reported in previous studies conducted in the same age group in Mozambique, which reported frequencies of 15% and 13.9% respectively [2,7]. Methodologically, our study differed from previous studies because for the first time highly sensitive qPCR was used, which explains the differences. In fact, similar findings were repeatedly reported in other countries [9,24,25]. Nowadays, it is widely known that sensitivity of microbiological culture in limited-resource settings is lower [8,14]. M-qPCR has the added advantage of providing results more rapidly. M-qPCR results are available within a few hours, whereas the culture method requires at least a full day before results can be determined [8,10]. In addition, M-qPCR is less affected by consumption of antibiotics prior to lumbar puncture [8] and amplification of DNA from non-viable bacteria improves identification in culture-negative samples [12,14,26–28]. Since Mozambique is currently expanding PCR methods for other infectious disease such as Tuberculosis and HIV at national and regional public health laboratories and also at the provincial laboratories findings of our study suggest that M-qPCR, if incorporated into routine public health surveillance at these referral laboratories, can improve the detection rate of bacterial meningitis in Mozambique and consequently improve estimates of the disease burden and provide better assessment of the impact of vaccinations.

The frequency of ABM in this study when using culture was 7%, which is much lower than the prevalence of ABM as reported in two previous studies conducted in Mozambique using similar method. Since prior studies were conducted before the introduction of vaccination for *H*. *influenzae* in 2009, we can hypothesise that introduction of the *H*. *influenzae* vaccine was responsible for the reduction of ABM. However, although the burden of *H*. *influenzae* reduced since the introduction of vaccine, we acknowledge that it frequency is still relatively high in our study. We believe that emergence of non-serotype b strain of *Haemophilus influenzae* might partially explain these values, because data from National Immunization Program in Mozambique show that *Hib* vaccine coverage is high [29]. Similar findings were also reported in other places, such as Brazil [30] and recently in Gambia [31]. But further studies should be conducted to confirm this hypothesis.

Results of this study showed that *S*. *pneumoniae* was the leading cause of childhood bacterial meningitis in Mozambique, responsible for more than half (62.7%) of all laboratory-confirmed ABM using M-qPCR. Our findings are in agreement with those previously described for children under 5 years of age in other countries in Sub-Saharan Africa [1,2,32,33].

The burden of pneumococcal meningitis in children under 5 years of age in many countries has dramatically decreased following introduction of the pneumococcal conjugate vaccine [34,35]. In Mozambique, PCV-10 was introduced very recently, and this is the reason likely why *S*. *pneumoniae* still remains the most common cause of childhood bacterial. However, this finding should also be interpreted with caution since we used *ply* gene for the molecular detection of pneumococci. Recent data demonstrated that *ply* gene is not 100% specific for pneumococci, as this gene can also be found in other streptococci [36,37]. We used this gene because our real time PCR was developed a few years ago and at that time *ply* was considered acceptable for the detection of *S*. *pneumoniae* by PCR. We are now designing a new primer set targeting *lytA* gene, which is now considered to be more specific for *S*. *pneumoniae* [36–38].

*N*. *meningitidis* was the third most common agent in this study, with relatively low prevalence. These findings are in accordance with previous studies conducted in Manhiça, a district situated in southern Mozambique [2], but differ from previous findings reported in other African countries [1], where the relative frequency of meningococcal meningitis has been shown to increase after the introduction of the *Hib* vaccine [39]. In Africa, affected countries included Burkina Faso, Benin, Chad, and Ghana [40].

The contribution of pathogens other than pneumococci, *H*. *influenzae*, and meningococci has been reported in many developing countries [41]. In this study we observed that group B streptococcus was identified in the majority of laboratory-confirmed ABM in newborn using M-qPCR (90.9%). Similar findings were reported in several studies conducted in other resource-limited settings [42–46].

In many countries, especially in Sub-Saharan Africa and Asia, group B streptococcus represents the main cause of bacterial meningitis in the neonates [47–49]. Neonatal meningitis caused by *S*. *agalactiae* is also associated with high morbidity and mortality in many countries worldwide [46,50,51], despite the availability of affordable preventive and therapeutic interventions [51–57].

We found that a significant number of CSF were negative for all of the investigated bacteria. This likely suggests that virus, parasites and bacteria, other than *S*. *agalactiae*, *S*. *pneumoniae*, *N*. *meningitidis*, and *H*. *influenzae* might be causing meningitis or meningo-encephalitis in children in Mozambique. For this reason is underway at our laboratory, the establishment of molecular for the detection of a large set of pathogens.

Regarding antimicrobial susceptibility profile, we found that isolates of *S*. *pneumoniae* were highly resistant to penicillin (88.2% of isolates) and trimethoprim/sulfamethoxazole (100% of isolates). These findings differ from the results of previous studies conducted in Mozambique that reported that 90% of pneumococci isolates were susceptible to penicillin [6], but are in agreement with findings of other studies conducted in Ethiopia, Kuwait [58], and Nigeria [58–60]. These differences might be attributable to differences in the study design, study timelines, and differences in the laboratory methodology between studies.

*Hib* and meningococci isolates were 100% susceptible to ceftriaxone. These results are in agreement with other studies conducted in many Sub-Saharan Africa countries [41,61,62].

Antibiotic prescriptions for meningitis therapy are dependent on antimicrobial susceptibility patterns [41]. Guidelines for ABM treatment recommends penicillin and chloramphenicol as the first choice [6] and ceftriaxone as an alternative [21]. In this context, the results of this study strongly suggest that ceftriaxone would be considered as the best choice for treatment of ABM in children.

We also investigated predictors for ABM in our study group and we found that Gram stain had a sensitivity of 71%. Gram staining in the CSF samples is considered one of the most important methods for the laboratory confirmation of bacterial meningitis. In addition, this method is rapid and inexpensive [63]. However, the operator’s skills affect the accuracy of this method. In our study, the competence of the laboratory staff in each study site (assessed prior to study initiation using proficiency panels provided by the Reference laboratory) likely contributed to the excellent performance of Gram staining. Therefore, this scenario might not be replicated exactly in other hospitals or health centres in Mozambique.

We also found that age of the children, leucocyte count, and glucose and proteins levels were also strongly associated with ABM, which is similar to the findings of other authors [6,33,64–66] and children below 12 months of age are 3.9 times at risk of acquiring ABM infection (*p* = 0.000).

Regarding seasonal variability, our data demonstrated that ABM increased from July through November, peaking in July. This is not surprising as the dry season provides conditions for the destruction the mucosal defences, thus making children more susceptible to meningitis [67–70]. In addition, the increased frequency of viral respiratory disease during the dry season contributes to this problem [71,72].

We would like to acknowledge some limitations of the current study, such as: 1) the number of patients recruited at each sentinel site was different, which represent a sampling bias. This might be attributed to differences in the demographics of the populations at each region, difference in the capacity of each hospital and differences in the efficiency to enrol patients 2) molecular typing of *H*. *influenzae* to investigate distribution of the serotypes of this bacteria was not performed and for this reason we were not able to discriminate type b and non type b strains of *H*. *influenzae*; 3) Molecular detection of pneumococci, was performed using *ply* gene which is not 100% specific for pneumococci; 4) the morphological information on the isolated bacteria was not provided because the standard case investigation form does not collect this data, but this form is under revision to include this information and 5) beta-lactamase testing on the *H*. *influenzae* isolates was not performed.

## Conclusion

In conclusion, our results showed that ABM is highly prevalent among children with meningitis and that *S*. *pneumoniae* was the most common cause of acute bacterial meningitis in children under 5 years of age. M-qPCR significantly increased the detection rate of *N*. *meningitidis*, *S*. *pneumoniae*, *H*. *influenzae*, and group B streptococci and for this reason, the implementation of M-qPCR assays for bacterial meningitis diagnosis is of paramount importance to overcome the disadvantages of culturing, that otherwise result in a high rate of misdiagnoses of ABM in Mozambique. Our study also suggests that in this age group, in Mozambique, ampicillin and ceftriaxone are the preferred antibiotics for the treatment of bacterial meningitis.

These results confirm the importance of ongoing national meningitis surveillance to provide the valuable information considered critical for evidence-based policy making.
